# Health informatics in current healthcare system: A review

**DOI:** 10.6026/973206300221766

**Published:** 2026-03-31

**Authors:** Simran Mishra, Sweta Singh

**Affiliations:** 1 Department of Public Health Dentistry, K.M Shah Dental College and Hospital, Sumandeep Vidyapeeth (Deemed to be University), Vadodara, Gujarat, India

**Keywords:** Health informatics, electronic health records (EHRs), digital health, artificial intelligence (AI)

## Abstract

Health informatics integrates information science, computing and digital technologies to support clinical care, healthcare administration and public health. Therefore, it is of interest to review the applications, benefits and challenges of health informatics in contemporary healthcare systems. This review focuses on major health informatics applications such as electronic health records, clinical decision support systems, telemedicine and artificial intelligence. Thus, we show that these technologies contribute to improved quality of care, enhanced patient safety and increased healthcare efficiency through better data management and decision support. Additionally, health informatics supports population health monitoring and healthcare planning. However, significant challenges persist, including lack of interoperability, increased clinician workload, cybersecurity risks, data privacy concerns and ethical governance issues. Addressing these barriers is essential to ensure the secure, equitable and sustainable adoption of health informatics within modern healthcare systems.

## Background:

Healthcare systems increasingly rely on digital technologies to manage rising service demands and support evidence-based clinical and administrative choice making [1]. Health informatics facilitates the systematic collection, integration and use of health data, enabling efficient healthcare delivery and population health management [2]. Electronic health records (EHRs) form the foundation of health informatics infrastructure by supporting standardized documentation and longitudinal continuity of care across healthcare settings [3]. Recent studies indicate that EHR-based interventions, such as clinical nudges, are associated with improvements in healthcare quality and patient outcomes [4]. However, increased EHR use has also been linked to clinician cognitive burden and burnout, emphasizing the importance of usability and workflow integration [5]. Interoperability remains a key challenge in digital health implementation. The adoption of Fast Healthcare Interoperability Resources (FHIR) standards has improved semantic interoperability and facilitated health information exchange between disparate systems [6]. Clinical support systems enhance clinical care by integrating patient-specific data with evidence-based recommendations [7]. The incorporation of artificial intelligence into CDSS has expanded their capacity to integrate electronic health records with patient-generated health data, supporting more informed clinical choice making [8]. Telemedicine and digital health technologies have expanded access to healthcare services, particularly for patients with chronic diseases and those in underserved or remote areas [9]. Ethical concerns related to artificial intelligence, including bias and accountability, further highlight the need for robust governance frameworks in health informatics [10]. Therefore, it is of interest to review available data on the use of Health Informatics in current healthcare systems.

## Applications of health informatics:

Health informatics applications encompass electronic health records, interoperability frameworks, clinical support systems and telemedicine platforms. EHR systems enable standardized documentation and continuity of care across healthcare settings [3]. Interoperability frameworks based on FHIR standards facilitate secure and efficient data exchange between healthcare organizations [6]. AI-enabled CDSS support clinicians by synthesizing complex clinical data to assist diagnostic and therapeutic choices [8]. Telemedicine platforms improve healthcare accessibility and support long-term disease management [9].

## Benefits of health informatics:

At the patient level, health informatics improves care coordination, safety and engagement through improved access to health information [4]. For healthcare professionals, timely access to integrated clinical data supports evidence-based practice, although excessive documentation demands may contribute to workload stress [5]. At the organizational level, health informatics systems enhance operational efficiency, quality monitoring and data-driven choice making [1]. At the population level, integrated health data supports surveillance, planning and health policy development [2].

## Challenges and ethical considerations:

Major barriers to effective health informatics implementation include limited interoperability, system usability challenges and workforce adaptation [6]. Clinician burnout related to EHR use remains a significant concern [5]. Additionally, ethical challenges associated with artificial intelligence-such as algorithmic bias, lack of transparency and unclear accountability-necessitate comprehensive ethical governance and regulatory oversight [10].

## Conclusion:

Health informatics enhances clinical decision-making, patient safety and healthcare efficiency through technologies like EHRs, telemedicine and AI. It supports data-driven planning and improved public health outcomes. However, challenges such as interoperability, cybersecurity and ethical concerns must be addressed for its optimal and secure use.
